# Clinical implications of host genetic variation and susceptibility to severe or critical COVID-19

**DOI:** 10.1186/s13073-022-01100-3

**Published:** 2022-08-19

**Authors:** Caspar I. van der Made, Mihai G. Netea, Frank L. van der Veerdonk, Alexander Hoischen

**Affiliations:** 1 Department of Internal Medicine and Radboud Center for Infectious Diseases (RCI), Radboud University Medical Center, Nijmegen, 6525 GA The Netherlands; 2 Department of Human Genetics, Radboud University Medical Center, Nijmegen, 6525 GA The Netherlands; 3 Radboud Institute of Molecular Life Sciences (RIMLS), Radboud University Medical Center, Nijmegen, 6525 GA The Netherlands; 4 Department for Immunology and Metabolism, Life and Medical Sciences Institute (LIMES), University of Bonn, Bonn, Germany

## Abstract

Since the start of the coronavirus disease 2019 (COVID-19) pandemic, important insights have been gained into virus biology and the host factors that modulate the human immune response against severe acute respiratory syndrome coronavirus 2 (SARS-CoV-2). COVID-19 displays a highly variable clinical picture that ranges from asymptomatic disease to lethal pneumonia. Apart from well-established general risk factors such as advanced age, male sex and chronic comorbidities, differences in host genetics have been shown to influence the individual predisposition to develop severe manifestations of COVID-19. These differences range from common susceptibility loci to rare genetic variants with strongly predisposing effects, or proven pathogenic variants that lead to known or novel inborn errors of immunity (IEI), which constitute a growing group of heterogeneous Mendelian disorders with increased susceptibility to infectious disease, auto-inflammation, auto-immunity, allergy or malignancies. The current genetic findings point towards a convergence of common and rare genetic variants that impact the interferon signalling pathways in patients with severe or critical COVID-19. Monogenic risk factors that impact IFN-I signalling have an expected prevalence between 1 and 5% in young, previously healthy individuals (<60 years of age) with critical COVID-19. The identification of these IEI such as X-linked TLR7 deficiency indicates a possibility for targeted genetic screening and personalized clinical management. This review aims to provide an overview of our current understanding of the host genetic factors that predispose to severe manifestations of COVID-19 and focuses on rare variants in IFN-I signalling genes and their potential clinical implications.

## Background

During the coronavirus disease 2019 (COVID-19) pandemic, our knowledge on the SARS-CoV-2 virus and its interaction with the human host has rapidly expanded. COVID-19 displays a notable inter-individual variability in clinical symptoms, ranging from asymptomatic disease to lethal pneumonia [1, 2]. Part of this inter-individual heterogeneity can be explained by differences in the host genetic profile, which can both increase susceptibility and confer protective effects [3]. These differences are determined by both common and rare genetic variants in the host genome. Large-scale genome-wide association studies (GWAS) are being undertaken to elucidate common genetic variation and generate valuable information on a population level by identifying loci that are enriched in patients with symptomatic or severe COVID-19. Such studies can provide important information on the biologic pathways important for a disease. On the other hand, classical GWAS cannot effectively detect rare or private genetic variation, which often characterizes patients with extreme phenotypes of the disease [3, 4].

Although disease severity is disproportionally higher among the elderly, men and individuals with chronic comorbidities, severe or critical cases of COVID-19 can also occur in younger, previously healthy individuals [1, 2, 5, 6]. These individuals might carry rare genetic variants with strongly predisposing effects, or mutations that lead to known or novel immunodeficiencies that significantly impair the immune pathways central to the defence against SARS-CoV-2 infection [3, 7–10]. The identification of such monogenic disorders can help to elucidate the mechanistic basis of the immunopathogenesis underlying severe COVID-19, as these inborn errors of immunity (IEI) enable scientists to study the effects of severe and specific dysfunctions of host defence. Moreover, genetic diagnosis and clinical counselling might facilitate preventive, diagnostic and therapeutic interventions. This review will summarize the current knowledge on host genetic variants that are associated with severe forms of COVID-19. We focus on rare genetic variants that have been shown to explain a significant proportion of cases with critical COVID-19 and discuss the potential clinical implications.

## Genetic susceptibility to coronavirus infections

Infectious diseases have shaped the human genome during evolution through pathogen-imposed selection pressures [11]. Genome-wide sequencing studies have shown that genes involved in immunity display strong selection patterns, thereby delineating genes and pathways crucial in host defence [11]. The observed inter-individual heterogeneity in the outcome of infectious diseases is in many cases due to rare or common variation of the human host genome that can result in a clinical spectrum ranging from rare Mendelian diseases to altered individual susceptibility to complex immune-related phenotypes such as COVID-19. Diverse genetic backgrounds might also explain the widespread differences in COVID-19 severity across peoples of varying genetic ancestries, as the prevalence of genetic risk factors in these populations could differ. These differences could have originated from distinct degrees of selection pressure exerted by specific infectious agents in previous outbreaks, such as recently exemplified for tuberculosis [12].

### Genetic associations in previous coronavirus outbreaks

Until the COVID-19 pandemic, six human-tropic coronaviruses had been described. These include the alphacoronaviruses HCoV-NL63 and HCoV-229E and betacoronaviruses HCoV-OC43, HCoV-HKU1, SARS-CoV-1 and Middle East respiratory syndrome (MERS), the first four of which are seasonal “common cold” viruses [13]. Although large, systematic and reproducible genetic studies to address the host genetic variation influencing the immune response against coronavirus infections before the COVID-19 pandemic are lacking, the SARS outbreak in 2003 has prompted numerous candidate-based association studies [13, 14]. Most of these have investigated a link between specific host genetic variants or loci that could affect the function of genes involved in the human antiviral innate immune response and susceptibility to develop SARS-CoV-1 infection or a relationship with clinical outcomes [14–20]. For example, specific single-nucleotide polymorphisms (SNPs) in interferon gamma (IFNγ) and chemokine CCL5 (RANTES) were demonstrated to significantly associate with SARS susceptibility and mortality [15, 16]. In addition, three large Chinese case-control studies have shown an association between polymorphisms in *MBL2* and SARS-CoV-1 susceptibility, leading to mannose-binding lectin (MBL) deficiency with impaired opsonophagocytic viral killing [17–19]. In patients with MERS, only one very small cohort study has described an association between the human leukocyte antigen (HLA) class II alleles HLA-DRB1*11:01 and HLA-DQB1*02:02 and disease susceptibility [21]. The human and animal host genetic variants that have been identified in previous coronavirus infections have been comprehensively reviewed elsewhere, although it should be stressed that no robust associations passing genome-wide significance thresholds have been found due to limitations of the study designs [20].

### Genetic associations with susceptibility to severe or critical SARS-CoV-2 infection

During the COVID-19 pandemic, large consortia such as the Severe COVID-19 GWAS group [22], the GenOMICC and ISARIC groups [23] and the COVID-19 Host Genetics Initiative (HGI; https://www.covid19hg.org) have made unparalleled efforts of team science and international data sharing to investigate the association of host genetic variants with SARS-CoV-2 infection by performing GWAS at an unprecedented scale (Table 1) [4]. The largest GWAS by HGI leveraged the genomic data from multiple clinical studies, existing biobank and cohort studies and data from consumer genetic companies to investigate genetic loci associated with disease susceptibility or severity [24]. Their main analyses include more than 2.5 million population controls and over 50,000 COVID-19 patients, categorized depending on disease severity and hospital setting; several genetic loci have been associated with disease susceptibility or severity. In the GWAS studies, severe COVID-19 has generally been defined as PCR-proven SARS-CoV-2 infection leading to death or respiratory insufficiency that required hospitalization with either non-invasive high-flow oxygen devices or invasive mechanical ventilation [24]. The first signals that were reported to be associated with severe disease included genetic loci on chromosome 3p21.31 and 9q34.2 [22, 25, 26]. The 3p21.31 cluster is the most robustly replicated signal in several studies with a twofold increased risk of respiratory failure from COVID-19 and is suggested to be inherited from Neanderthals [22, 27]. Subsequently, various other genetic loci have reached genome-wide significance along with increasing sample sizes (Table 1) [23, 24, 28]. Several of these loci implicate genes in proximity to the lead SNP that encode proteins that regulate either the antiviral or the pro-inflammatory (organ-specific) host response. Examples of antiviral pathways targeted by the first group involve SARS-CoV-2 cell entry via interaction of ACE2 (*SLC6A20*), type I interferon (IFN) signalling (*IFNAR1*, *IFNAR2* and *RAVER1*), IFN-induced restriction of viral replication (*OAS1*, *OAS2* and *OAS3*), IL-10 and type III IFN signalling (*IL10RB*) [22, 26, 29, 30]. Furthermore, the GWAS results point to several genes that are expressed in pulmonary epithelial cells with distinct functions (*LZTFL1*, *DPP9*, *ELF5*, *MUC5B*, *FOXP4*, *SFTPD*), underlining the importance of these non-immune genes to the innate antiviral response [24]. Examples of pro-inflammatory pathways implicated by the GWAS results involve the activation of cytokine receptor signalling (*TYK2*), receptors that facilitate chemotaxis of immune cells to the infected tissue (*CCR1*, *CCR2*, *XCR1*, *CXCR6* and *CCR9*) and inflammasome activation (*DPP9*); this dipeptidyl peptidase has also been associated with lung fibrosis and belongs to the DPP4 family, where DPP4 is the entry receptor for MERS [22, 26, 29, 31, 32].

**Table 1:** Significant large-scale genome-wide associations in patients with severe or critical COVID-19

GWAS study	Study participants	Controls	Genetic ancestry	Genetic cluster(s)	Lead SNP(s) or variant	Gene(s) implicated
*Ganna et al.* COVID-19 Host Genetics Initiative (meta-analysis of 46 studies, including the update following data release 6 (www.covid19hg.org)) [24, 33]	COVID-19 patients who required respiratory support or who died as a consequence of COVID-19 *N* = 9376	Population controls *N* = 1,776,645	European, Admixed American, African, Middle Eastern, South Asian and East Asian	1p21.3	rs67579710	i.e. *THBS3*, *MUC1*, *EFNA1*
				3p.21.31	rs35508621	*LZTFL1*, *CXCR6*
				6p21.33	rs111837807	*HLA-B*, *HLA-C*
				6p21.1	rs1886814	*FOXP4*
				9q34.2	rs912805253	*ABO*
				12q24.13	rs10774679	*OAS1*, *OAS2*, *OAS3*
				12q24.33	rs12809318	*FBRSL1*
				16q24.3	rs117169628	i.e. *SLC22A31*, *ACSF3*
				19p13.3	rs2109069	*DPP9*
				19p13.2	rs74956615	*TYK2*
				21q22.1	rs13050728	*IFNAR2*, *IL10RB*
	Patients with moderate or severe COVID-19 who required hospitalization *N* = 25,027	Population controls *N* = 2,836,272	European, Admixed American, African, Middle Eastern, South Asian and East Asian	1p21.3	rs67579710	i.e. *THBS3*, *MUC1*, *EFNA1*
				3p.21.31	rs73062389 rs35508621	*SLC6A20*, *SACM1L* *LZTFL1*, *CXCR6*
				6p21.33	rs111837807	*HLA-B*, *HLA-C*
				6p21.1	rs1886814	*FOXP4*
				9q34.2	rs912805253	*ABO*
				10q22.3	rs721917	*SFTPD*
				11p15.5	rs35705950	*MUC5B*
				11p13	rs766826	*ELF5*
				12q24.13	rs10774679	*OAS1*, *OAS2*, *OAS3*
				12q24.33	rs12809318	*FBRSL1*
				19p13.3	rs2109069	*DPP9*
				19p13.2	rs74956615	*TYK2*
				21q22.1	rs13050728	*IFNAR2*, *IL10RB*
*Ellinghaus et al.* The Severe COVID-19 GWAS Group [34]	COVID-19 patients hospitalized at the general ward or ICU with respiratory failure, defined as patients requiring oxygen supplementation (non-invasive) or mechanical ventilation. *N* = 1980	Population controls *N* = 2381	European	3p.21.31	rs11385942	*SLC6A20*, *LZTFL1*, *FYCO1*, *CXCR6*, *XCR1* and *CCR9*
				9q34.2	rs657152	*ABO*
*Shelton et al.* 23andMe [26]	Patients with self-reported COVID-19 and hospitalization, pneumonia and/or respiratory support *N* = 1447	Population controls *N*= 796,151	European, Latino, African American	3p.21.31	rs13078854	*SLC6A20*, *LZTFL1*, *FYCO1*, *CXCR6*, *XCR1* and *CCR9*
*Pairo-Castineira et al.* GenOMICC/ISARIC [23]	COVID-19 patients requiring continuous cardiorespiratory monitoring, in high-dependency or intensive care units *N* = 2244 (primary analysis on data from 1676 individuals of European ancestry)	Ancestry-matched population controls *N* = 8380	European, South Asian, African, East Asian	3p.21.31	rs73064425	*LZTFL1*, *CXCR6*, *FYCO1*, *CCR3*
				6p22.1	rs9380142	*HLA-G*^*a*^
				6p21.33	rs143334143	*CCHCR1*^*a*^
				6p21.32	rs3131294	*NOTCH4*^*a*^
				12q24.13	rs10735079	*OAS1*, *OAS2*, *OAS3*
				19p13.3	rs2109069	*DPP9*
				19p13.2	rs74956615	*TYK2*
				21q22.1	rs2236757	*IFNAR2*
*Andolfo et al.*^b^ [28]	Hospitalized COVID-19 patients *N* = 7970	Population controls *N* = 902,088	European	21q22.1	rs13050728	*IFNAR2*
				21q22.3	rs3787946	*MX1*, *TMPRSS2*
*Kousanathos et al.*^c^ GenOMICC/ISARIC [35]	COVID-19 patients requiring continuous cardiorespiratory monitoring, in high-dependency or intensive care units *N* = 7491	Ancestry-matched population controls *N* = 48,400 controls	European, South Asian, African, East Asian	1p21.3	rs114301457, rs7528026, rs41264915	*MUC1*, *THBS3*, *EFNA4, TRIM46*
				2p16.1	rs1123573	*BCL11A*
				3p.21.31	rs2271616, rs73064425	*LZTFL1*, *CXCR6*, *CCR9*, *XCR1*
				6p21.33	rs9271609	*HLA-G*, *HLA-DRB1*, *HLA-DQA1*
				11p13	rs61882275	*ELF5*
				12.q24.33	rs56106917	*FBRLS1*
				13q34	rs9577175	*ATP11A*
				19p13.3r	s12610495	*DPP9*
				19p13.2	rs34536443	*TYK2*
				21q22.1	rs17860115, rs8178521	*IFNAR2*, *IL10RB*, *IFNAR1*

^a^The authors state that these variants are located in a region of chromosome 6 for which population stratification is difficult to control (the major histocompatibility complex) and did not replicate in a meta-analysis of data from other studies

^b^The authors have conducted a meta-analysis on existing data from the COVID-19 Human Genetic Effort using summary statistics, with a focus on chromosome 21. The investigated 21q22.3 locus did not harbour genome-wide significant eQTLs (*p*-value ≤ 5 × 10^−8^); however, 5 common SNPs in this locus (*p*-value ≤ 1 × 10^−5^) were validated in other cohorts of hospitalized COVID-19 patients

^c^This study involved critical COVID-19 patients that were recruited to the GenOMICC study, of which 1339 had already been included in a primary analysis published previously [23]

Follow-up studies have already provided evidence towards likely causality for specific associations through fine-mapping and functional experiments. For example, a specific variant (rs17713054G>A) in tight linkage disequilibrium with the lead SNPs in the 3p21.31 cluster was demonstrated to be an enhancer motif variant that increased the expression of *LZTFL1* [36]. The authors hypothesize that the repressive activity of LZTFL1 on the epithelial–mesenchymal transition of pulmonary epithelial cells, a process that limits viral infection in the acute setting but is also upregulated in spatial transcriptomic analysis of lung biopsies from patients with fatal COVID-19, could explain the elevated risk attributed to the 3p21.31 cluster. For two other genes located within the 3p21.31 cluster, the cytokine receptor encoding *CCR1* and *CXCR6*, it was shown that their expression on CD8^+^ T cells and monocytes correlated with COVID-19 severity by combining GWAS and single-cell RNA sequencing data [37]. Furthermore, two recent studies have implicated *OAS1* as the suspected causal gene in the 12q24.13 locus that contains the OAS gene cluster, showing that the risk haplotype correlated with two variants that affected splicing and decreased nonsense-mediated decay of *OAS1* transcripts [38], which impaired OAS1 antiviral activity [39]. A detailed functional study further elucidated that one of these variants, rs10774671(G>A), impaired C-terminal prenylation of the OAS1 isoforms that consequently do not efficiently detect SARS-CoV-2, while higher concentrations of circulating OAS1 and expression of prenylated OAS1 have been associated with protection from severe COVID-19 [40].

Furthermore, an association was found between the *ABO* blood type locus on chromosome 9q34.2 and COVID-19 susceptibility [22–24, 26, 41]. In support, several observational cohort studies have reported a protective effect of blood group O for developing critical COVID-19, as opposed to non-O blood groups [22, 42–45]. Previous work has shown that ABO blood groups can play direct roles as pathogen (co-)receptors and that genetic variation in the *ABO* locus is associated with disease susceptibility to various infectious agents such as SARS-CoV-1 [46]. In addition, differences in blood type are directly linked to haemostasis and endothelial integrity, as shown by genetic associations with cardiovascular disease, venous thromboembolism and plasma levels of von Willebrand factor [42, 47]. The consequences of the variable blood type functions might be especially important in COVID-19, as endothelitis and coagulopathy are cardinal features of severe disease. These data demonstrate that genetic findings both confirm the epidemiological observation and link them to the pathophysiology of the disease.

The data generated by these GWAS studies have already provided essential information on genetic loci conferring susceptibility to or protection from SARS-CoV-2 infection or an increased risk for severe disease. Most insights for loci, genes or pathways are generated on a population level and have taught important lessons about the general pathophysiology of COVID-19. GWAS studies could even identify highly penetrant common variants, as recently shown for tuberculosis [12]. The study of rare variants is another approach to investigate such host genetic factors that strongly increase an individual risk for disease susceptibility or severity. In comparison, even the most robustly associated and replicated common variants are predicted to modestly increase an individual’s risk for severe COVID-19 by a maximum odds ratio (OR) of about 2 [32], as opposed to an estimated OR of 50 for rare variants that cause IEI [48]. Three studies have so far performed large-scale genome- or exome-wide studies that studied associations between rare variants and disease susceptibility in non-hospitalized patients with confirmed SARS-CoV-2 infection compared to population controls [41, 49, 50]. Only one rare variant upstream of *ACE2* was found that reached exome-wide significance, correlating with decreased expression of the SARS-CoV-2 receptor [41]. Most studies on rare variants have however focused on the identification of monogenic factors enriched in cohorts of patients with life-threatening disease and using a candidate gene-based approach [8, 51–55]. Moreover, in addition to the standard GWAS that assesses individual variant associations, an approach that by design has a limited power to detect individual rare variants, weighed burden tests have been performed to validate enrichment of rare variants in specific genes in patients. The next sections will focus on our current understanding of rare genetic mutations or IEI that predispose to severe or critical COVID-19.

## Inborn errors of immunity (IEI) predisposing patients to severe or critical COVID-19

Severe or critical cases of COVID-19 have also been reported in individuals below 50 years of age who were previously healthy. Since the risk of critical COVID-19 is most significantly correlated with age, its manifestation in relatively young patients could indicate the presence of strong predisposing genetic variants that significantly impair the core immune pathways engaged in the defence against SARS-CoV-2 infection. Additionally, elderly individuals could be identified with similar genetic variants that until now had been redundant in their immune response, but became manifest in the setting of SARS-CoV-2 infection. While these predisposing host genetic variants may be individually rare and explain a minority of severe or critical COVID-19 cases, their identification can highlight central mechanisms in the pathogenesis of COVID-19 and lead to personalized patient management. The next sections will discuss the current literature on known and novel IEI in patients with COVID-19.

### COVID-19 in patients with known inborn errors of immunity

Several studies have documented outcomes of SARS-CoV-2 infection in patients with known IEI, ranging from case reports and single-centre case series to larger, multicentre cohort studies [56]. To our knowledge, a total of 9 larger studies have been conducted that collectively report on 545 IEI patients; the findings of these studies have been summarized in Table 2 [57–65]. The majority of IEI groups that were defined based on the International Union of Immunological Societies (IUIS) classification were represented across these cohorts [66, 67]. The largest groups comprised patients with antibody deficiencies and combined immunodeficiencies.

**Table 2:** An overview of reported outcomes of SARS-CoV-2 infection in patients with known inborn errors of immunity

	Delavari et al. [63]	Marcus et al. [68]	Hsi-en Ho et al. [64]	Meyts et al. [57]	Shields et al. [69]	Castano-Jaramillo et al. [70]	Esenboga et al. [65]	Millito et al. [71]	Goudouris et al. [72]	Total
Demographics										
Genetic ancestry	Iran	Israel	USA	Europe, UK, Latin America, USA	UK	Mexico	Turkey	Italy	Brazil	
Age, mean (IQR)	5.19 (1.04–8.96)	14.3 (15–30)	44.5 (28.0–64.0)	25–34^b^	42.0 (28.0–57.0)	22.5 (11.5–29.5)	20.5 (9.41–39.0)	35.3	25.1 (10.7–38.6)	
<18y, %	78.9	45	12.5	34	7.5	51.6	42.3	25.2	47.1	
>18y, %	21.1	55	87.5	66	92.5	48.4	57.7	74.8	52.9	
Sex, M:F	63:37	60:40	69:31	65:35	57:43	74:28	54:46	61:39	45:55	
Distribution of IEI groups										
Total, *n*	19	20	16	94	67^c^	31	26	131	121^e^	525
Primary antibody deficiency (PAD), *n* (%)	4 (21.1)	6 (30.0)	14 (87.5)	53 (56.4)	45 (67.2)	20 (64.5)	13 (50.0)	99 (75.6)	53 (43.8)	307 (58.5)
CVID	1	4	9	29	23	11	5	76	26	184
Agammaglobulinemia (XL/AR)	1	2	3	6	4	7	4	16	11	54
Other (hypogammaglobulinemia, specific Ab or Ig subclass deficiency)	2	0	2	18	18	2	4	7	16	69
Combined immunodeficiency, *n* (%)	10 (52.6)	9 (45)	1 (6.3)	14 (14.9)	4 (6.0)	3 (9.7)	7^d^ (26.9)	22 (17.0)	17 (14.0)	87 (16.6)
Syndromal	4	1	0	10	3	1	6	14	5	44
Non-syndromal (including SCID)^a^	6	8	1	4	1	2	1	8	12^f^	31
Immune dysregulation, *n* (%)	2 (10.5)	3 (15)	0 (0)	9 (9.6)	4 (3.0)	1 (3.2)	4 (15.4)	2 (1.5)	3 (2.5)	28 (5.3)
EBV/HLH	2	0	0	1	2	0	1	0	3	9
Autoimmunity	0	3	0	8	2	1	3	2	0	19
Auto-inflammatory disorder, *n* (%)	1 (5.3)	0	0 (0)	7 (7.4)	3 (4.5)	1 (3.2)	0 (0)	1 (0.8)	9 (7.4)	22 (4.2)
Periodic fever syndrome	0	0	0	3	1	0	0	0	6	10
Interferonopathy	0	0	0	3	1	0	0	1	0	5
Other	1	0	0	1	1	1	0	0	3	7
Phagocyte defect, *n* (%)	2 (10.5)	2 (10)	0 (0)	6 (6.4)	4 (4.5)	5 (16.1)	1 (3.8)	0 (0)	6 (5.0)	26 (5.0)
Functional defect (i.e. CGD)	2	2	0	4	4	5	0	0	5	22
Neutropenia/other	0	0	0	2	0	0	1	0	1	4
Innate/intrinsic defect, *n* (%)	0 (0)	0 (0)	1 (6.3)	3 (3.2)	0 (0)	0 (0)	1 (3.8)	4 (3.1)	7 (5.8)	16 (3.0)
Bacterial/parasitic	0	0	1	2	0	0	1	2	4	10
MSMD/viral	0	0	0	1	0	0	0	2	3	6
Complement deficiencies, *n* (%)	0 (0)	0 (0)	0 (0)	0 (0)	5 (7.5)	0 (0)	0 (0)	0 (0)	25 (20.7)	30 (5.7)
Bone marrow failure, *n* (%)	0 (0)	0 (0)	0 (0)	2 (2.1)	0 (0)	0 (0)	0 (0)	0 (0)	0 (0)	2 (0.4)
Phenocopies, *n* (%)	0 (0)	0 (0)	0 (0)	0 (0)	0 (0)	1 (3.2)	0 (0)	3 (2.3)	1 (0.8)	5 (1.0)
**COVID-19 outcomes**										
Hospitalization, %	100	0	75	63	50.7	48	38.4	NA	28.9	184/394 (46.7)
Respiratory insufficiency, %	NA	0	62.5	31	NA	NA	15.3	NA	18.1	68/266 (25.6)
Mechanical ventilation, %	NA	0	31.3	16	NA	NA	7.7	NA	NA	
(N)ICU admission, %	42.1	0	31.3	21.2	NA	26	7.7	NA	NA	
Infection fatality rate (%)	8/19 (42.1)	0	4/16 (25)	9/94 (9.6)	12/67 (17.9)	6 (19.4)	2/26 (7.7)	5/131 (3.8)	6/121 (5.0)	52/525 (9.9)
Underlying diagnoses among fatal cases	STK4, RAB27, DNMT3B and IL1RN deficiency, SCID (*n*=4)	NA	CVID (*n*=2), hypogammaglobulinemia, IgA-IgG2 deficiency	X-CGD with HLH, XIAP deficiency with GVHD following HSCT and septic shock/HLH, syndromic IEI with heart failure, pulmonary hypertension and a pneumothorax, and antibody deficiencies (CVID *n*=4, IgG *n*=1, IgA/IgG2 *n*=1)	Primary antibody deficiency (CVID (*n*=8), unclassified PAD (*n*=1), polysaccharide antibody deficiency (*n*-1), unclassified CID (*n*=1), CTLA4 (*n*=1))	Four children died (WAS, XLA with secondary HLH, CGD and unspecified auto-inflammatory syndrome both with MIS-C) and two adults (good syndrome and XLA with both bacterial superinfection)	LRBA deficiency, EBV-related NHL receiving chemotherapy	NA	XLA (*n*=2), CVID, hyper IgM syndrome/CD40L deficiency, good syndrome and XIAP deficiency	
(IEI-associated) comorbidities among fatal cases	Pre-existing IEI-associated autoimmune/inflammatory complications in 3/8 patients, lymphoproliferation in 5/8	NA	Pre-existing IEI-associated autoimmune/inflammatory complications in 3/4 fatal cases, chronic lung disease in 2 and a previous kidney transplant in 1 patient	All had pre-existing comorbidities (cardiomyopathy, kidney transplant recipient with several malignancies, chronic lung and heart disease, diabetes, older age)	Patients were older and had more chronic comorbidities (chronic lung disease, chronic kidney disease, diabetes mellitus)	The WAS patient was post-HSCT and had a CMV infection and chronic lung disease, two patients had bronchiectasis, one had previous autoimmune disease, one had a chronic osteomyelitis	The patient with LRBA deficiency had pre-existing IEI-associated autoimmune disease and bronchiectasis	Patients were older and had pre-existing comorbidities in 2 of 5 patients (hypertension, obesity)	Disease severity correlated with age, immunoglobulin use and the number and type of comorbidities (bronchiectasis, cardiopathy) and inversely correlated with use of immunomodulatory treatment. There was no clear correlation between IEI group and severity of infection	

*Abbreviations*: *Ab* antibody, *Ig* immunoglobulin, *CGD* chronic granulomatous disease, *CMV* cytomegalovirus, *CVID* common variable immunodeficiency, *EBV* Epstein-Barr virus, *GVHD* graft versus host disease, *HLH* haemophagocytic lymphohistiocytosis, *HSCT* haematopoietic stem cell transplantation, *IQR* interquartile range, *MSMD* Mendelian susceptibility to mycobacterial disease, *NHL* non-Hodgkin lymphoma, *SCID* severe combined immunodeficiency, *XLA* X-linked agammaglobulinemia, *WAS* Wiskott-Aldrich syndrome

^a^The non-syndromal combined immunodeficiencies comprise the severe combined immunodeficiencies and the less profound combined immunodeficiencies

^b^The study cohort was stratified in distinct age groups; therefore, no exact mean can be calculated

^c^The complete cohort additionally included 33 patients with a secondary immunodeficiency

^d^The authors classify STAT1 GoF as CID, but in the most recent IUIS classification, this is classified as a defect in intrinsic and innate immunity

^e^Four patients (two with familial Mediterranean fever and two with CVID) were already reported in the study of Meyts et al. [57]

^f^Ten patients with SCID (*n* = 7), LAD (leukocyte adhesion deficiency) type III (*n* = 1), WAS (*n* = 1) and *XIAP* mutation (*n* = 1) had SARS-CoV-2 infection after haematopoietic stem cell transplant (HSCT)

Although a small majority of reported IEI patients with documented SARS-CoV-2 infections was either asymptomatic or only developed mild disease, 47% of patients required hospitalization [57, 63–65, 68–70, 72]. These studies have indicated that patients with monogenic IEI are at an increased risk of developing severe or critical COVID-19 although this increase for the whole group is modest compared to the general population [9, 73]. In the study by Meyts et al. [57], especially younger men were at a higher risk of developing severe COVID-19, possibly reflecting concurrent biological sex differences in the antiviral immune response [74]. Also, IEI patients contracted severe or fatal COVID-19 at a significantly younger age [57]. Since having an IEI does not seem to be an independent risk factor for severe or critical COVID-19, this could indicate a redundancy in different components of host defence mechanisms, as well as reflect effective treatment of the underlying IEI. For example, it can be speculated that the humoral immune response is not essential to resolve SARS-CoV-2 infection as most patients with antibody deficiencies developed mild or asymptomatic disease. However, most patients with antibody deficiencies were on immunoglobulin treatment, and a report of a patient with untreated common variable immunodeficiency (CVID) who developed fatal COVID-19 suggests that supplementation of IgG could be indispensable [75]. Additionally, many severe combined immunodeficiency (SCID) patients in the included studies, with both cellular and humoral defects due to the lack of B and T cells, had already underwent curative stem cell transplantation [57, 65, 71, 72].

Most studies assessing the immunological profile of patients with severe COVID-19 point towards an initial low-responsiveness state elicited by SARS-CoV-2, with defective or delayed innate and intrinsic antiviral type I/III interferon responses, progressing to virus-mediated tissue damage with an ensuing dysregulated hyperinflammatory response characterized especially by high levels of IL-1 and IL-6 [76–80]. It could therefore be hypothesized that IEI patients with an impaired antiviral response or patients with immune dysregulation, hyperinflammation or auto-inflammatory disorders, could be at an elevated risk at developing severe disease. Stratification of patients according to IEI group indicates that patients with immune dysregulation, and also combined immunodeficiencies (CID), associate with severe COVID-19 [63, 64, 81]. Patients with IEI-associated autoimmune or inflammatory complications were also at an elevated risk [62–64]. As patients with an innate (antiviral) immune defect constituted only 3% of IEI patients in the discussed studies [71], this group could not be studied in detail. Multiple prospective studies have however identified IEI of innate type I interferon (IFN-I) signalling as strong monogenic risk factors for severe or critical COVID-19. We will continue by focusing on these known and novel IEI affecting IFN-I signalling in the next two paragraphs. Moreover, we will briefly address the genetic predisposition to multisystem inflammatory syndrome in children (MIS-C), a distinct complication of COVID-19 in children and young adults.

### Inborn errors of type I interferon signalling in patients with severe or critical COVID-19

Early in the pandemic, the COVID-19 Human Genetic Effort (www.covidhge.com) was created to investigate the genetic and immunological determinants of critical COVID-19 [3]. This international consortium sequenced the exome or genome of patients with life-threatening COVID-19 pneumonia and of individuals with asymptomatic or mild infection. As a first approach, it was investigated whether genetic variants in IEI genes previously associated with life-threatening influenza pneumonia were enriched among patients with critical COVID-19 [51]. The authors therefore assessed the presence of rare variants in 13 genes known to affect the Toll-like receptor 3 (TLR3)– and interferon regulatory factor 7 (IRF7)–dependent type I interferon (IFN) immunity pathways in 659 patients with life-threatening COVID-19, defined as critical disease with respiratory insufficiency requiring mechanical ventilation or high-flow oxygen, septic shock or other organ damage requiring critical care in ICU, compared to 534 individuals with asymptomatic or mild SARS-CoV-2 infection [51]. In the exomes of 3.5% of patients, the authors identified rare genetic variants in 8 of the 13 genes in the TLR3- and IRF7-pathway (*IRF7*, *IFNAR1*, *IFNAR2*, *TLR3*, *TICAM1*, *TBK1*, *IRF3* and *UNC93B1*) that were shown to lead to loss-of-function (LoF) by functionally abolishing type I interferon signalling. The authors reported a total of 23 variants in these genes, of which 7 (30.4%) in the genes *UNC93B1*, *IRF7*, *IFNAR1* and *IFNAR2* followed AD inheritance that diverged from the established AR inheritance associated with severe influenza pneumonia [51]. Specific experiments were carried out to assess the altered immune response upon in vitro SARS-CoV-2 infection, showing that TLR3^−/−^, TLR3^+/−^, IRF7^−/−^ and IFNAR1^−/−^ fibroblasts had increased infection susceptibility and that plasmacytoid dendritic cells (pDC) from IRF7-deficient patients did not produce IFN-I after infection. The individuals carrying these deleterious variants had never been previously hospitalized for life-threatening viral illnesses and were aged between 17 and 77 years, suggesting that the penetrance of these variants for severe SARS-CoV-2 infection is higher compared to severe influenza pneumonia.

Several subsequent studies have replicated part of these findings [82–85]. Two children from Canada and Alaska that respectively developed recurrent severe COVID-19 or fatal COVID-19 were shown to harbour the same homozygous mutation in *IFNAR2* (p.(Ser53Pro)) [82]. Both patients had a history of disseminated viral infections after MMRV (measles, mumps, rubella and varicella) vaccination, unlike the two patients with AR IRF7 deficiency reported by the HGE cohort [51]. The identified *IFNAR2* variant occurred at a relatively high allele frequency in their Inuit ancestry and was demonstrated to lead to abrogated cell surface expression of IFNAR2 and diminished IFN-I signalling. Moreover, two additional children with AR IFNAR1 deficiency were described in separate case reports [83, 84]. A 3-year-old girl with both critical COVID-19 and fatal MIS-C had a homozygous large deletion leading to a frameshift that was confirmed to lead to LoF in HEK293T cells [84], while a 14-year-old boy with critical COVID-19 harboured a homozygous splice site variant previously reported as deleterious [83]. Lastly, in a child who developed fatal COVID-19, both a homozygous canonical splice site variant in *TBK1* and a homozygous missense variant in *TNFRSF13B*, a known risk factor associated with CVID, were identified [85]. The child had a history of unclassified auto-inflammation, which could be explained by the homozygous variants in *TBK1*, as it has recently been shown that AR TBK1 deficiency underlies TNF-driven systemic auto-inflammation [86]. Immunosuppressive treatment with prednisolone and methotrexate up until hospital admission might have contributed to the severe disease course.

Although a relationship between rare variants in these type I IFN genes and critical COVID-19 is plausible given the importance of intact type I IFN signalling in the anti-SARS-CoV-2 host immune response and the corroboration by some additional cases, four larger, independent studies failed to replicate the enrichment of variants in the TLR3 and IRF7-pathway in patients with critical COVID-19 [35, 49, 50, 55]. This discrepancy could be in part explained by differences in cohort characteristics, including age distribution, definition of disease severity and possibly also clinical baseline parameters such as pre-existent comorbidities or history of infections, and the use of a- or pauci-symptomatic versus population controls. Moreover, the determination of variant enrichment was based on in vitro biochemical and immunological experiments in the COVID-19 HGE study, while rare variant associations were assessed in silico with gene burden testing in the other studies. Lastly, no correction for differences in ancestry was performed in the COVID-19 HGE study [55]. Therefore, the exact prevalence and contribution of similar variants that affect TLR3- and IRF7-mediated IFN I signalling in a general cohort of patients with critical COVID-19 remain unclear and will be more definitively determined in studies with larger WES and whole-genome sequencing (WGS) datasets with more powerful group comparisons. Furthermore, it would be of interest to perform gene burden tests for AD and AR inheritance, considering that the mutations in known IEI genes associated with critical COVID-19 followed different inheritance patterns compared to life-threatening influenza pneumonia.

### A novel inborn error predisposing to severe COVID-19: X-linked TLR7 deficiency

In addition to the rare variants in known IEI that were associated with critical COVID-19, using an unbiased approach, we identified X-linked TLR7 deficiency as the first novel immunodeficiency in patients with critical COVID-19 [8]. In two unrelated young brother pairs with critical COVID-19, rare genetic variants in the X-chromosomal Toll-like receptor 7 (*TLR7*) were identified by rapid clinical whole-exome sequencing (WES). *TLR7* encodes an evolutionary highly conserved cytosolic pattern recognition receptor that recognizes single-stranded RNA viruses such as coronaviruses [87]. It had previously been shown that mice deficient in either *TLR7* or the downstream adaptor *MyD88* displayed impaired production of IFN-I, delayed viral clearance and severe lung pathology upon infection with MERS-CoV [88–90]. TLR7 is most abundantly expressed on plasmacytoid dendritic cells (pDCs), which are important producers of type I IFN. In peripheral blood cells isolated from these young male patients, the *TLR7* variants were shown to lead to an absence of the transcriptional IFN-I response and the production of interferon gamma (IFNγ) in response to a TLR7-specific agonist [8].

These preliminary findings describing X-linked TLR7 deficiency (OMIM #301051) in patients with critical COVID-19 have subsequently been replicated in several other cohorts (Table 3) [50, 53, 91]. Fallerini et al. described rare, hypomorphic or LoF *TLR7* missense variants in 3 out of 135 (2.2%) male patients with severe COVID-19 aged below 60 years of age [52]. In a larger cohort of 1202 patients with life-threatening pneumonia aged below 60 years of age from the COVID-19 HGE, X-linked TLR7 deficiency was diagnosed in 17 (1.4%) patients but not in the 331 male controls [53]. Additionally, 3 of 252 (1.1%) patients with severe COVID-19 defined as hospitalization with low-flow oxygen (<6 l/min) that were also included in the HGE cohort carried deleterious TLR7 variants. Importantly, it was observed that the clinical penetrance of these variants in patients with severe or critical pneumonia was high, but not complete, as three hemizygous relatives only had asymptomatic, mild or moderate disease symptoms. Incomplete penetrance was also suggested by another study [91]. The authors showed that EBV-immortalized B cell lines and myeloid cell subsets from these patients were irresponsive to stimulation with TLR7 agonists, which could be rescued by transfecting wild-type *TLR7*. Also, patient pDCs produced low amounts of type I IFNs in response to SARS-CoV-2. This study further established the key role of TLR7 signalling in the host defence against SARS-CoV-2 infection. It has also been demonstrated in nasal tissue, bronchoalveolar lavage and peripheral blood mononuclear cells extracted from COVID-19 patients that expression levels of *TLR7* correlated with disease severity [92, 93].

**Table 3:** Rare monogenic variants identified in patients with severe/critical COVID-19 or MIS-C

Affected gene	Inheritance pattern	Mutational mechanism	Functional defect	Severe/critical COVID-19 population(s) studied	Prevalence of proven defect(s)
Severe COVID-19					
*TLR7*	XLR	LoF (complete or hypomorph)	Impaired viral clearance due to disrupted TLR7 signaling with a defective production of type I/II interferons	Adult male brother pairs <35 years without predisposing comorbidities, who developed respiratory insufficiency requiring mechanical ventilation in ICU [8]	-
				Adult men aged <60 years with respiratory insufficiency requiring mechanical ventilation in ICU	2.2% (3/135)
				Adult men aged <50 years without predisposing comorbidities, who developed respiratory insufficiency requiring high-flow oxygen devices or mechanical ventilation [52]	14.3% (2/14)
				Individuals (*n* = 1304) with respiratory insufficiency requiring mechanical ventilation or resulting in death [50]	0.55% (7/1267)
				Men with life-threatening pneumonia requiring high-flow oxygen devices or mechanical ventilation, septic shock or another type of organ damage requiring ICU admission [53]	1.3% (16/1202)
*IRF7*	AD, AR	LoF (complete or hypomorph)	Impaired viral clearance due to defective type I interferon signaling and production	Men with life-threatening pneumonia requiring high-flow oxygen devices or mechanical ventilation, septic shock or another type of organ damage requiring ICU admission [51]	3.5% (24/659)
*TLR3*	AD				
*TICAM1*	AD				
*IRF3*	AD				
*TBK1*	AD				
*IFNAR1*	AD, AR				
*IFNAR2*	AD				
*UNC93B1*	AD				
MIS-C					
*SOCS1*	AD	LoF	Hyperinflammation due to decreased negative regulation of type I/II interferon signaling (increased STAT1 phosphorylation and expression of type I/II IFN-stimulated and proaptotic genes)	Children meeting the criteria for multisystem inflammatory syndrome (MIS-C), defined as fever, elevated inflammatory marker levels, multisystem organ involvement, and SARS-CoV-2 infection or exposure within 4 weeks of symptoms without an alternative diagnosis [54, 94]	17% (3/18)
*XIAP*	XLR	LoF	Hyperinflammation due to decreased negative regulation of the NLRP3 inflammasome with basally elevated levels of IL-6, IL-18, IL-10 and CXCL9 (and IL-1β after stimulation)		
*CYBB*	XLR	LoF	Hyperinflammation due a decreased phagocytic oxidative burst (impaired function of NADPH oxidase) and decreased inhibition of type I interferon signaling		

Furthermore, in pan-ancestry whole-exome sequencing data of over 500,000 individuals from the UK biobank, including more than 20,000 patients who contracted COVID-19, the burden of rare variants in *TLR7* was found to be significantly increased in patients with severe COVID-19 (OR 4.53 (2.64–7.77)) [50]. This analysis did not investigate sex-specific effects as suggested for X-linked traits, suggesting that the effect would be higher among men. In addition, another very recent analysis of the HGI WES/WGS data of 5048 severe disease cases and over 571,000 controls found a significant enrichment of rare deleterious *TLR7* variants in cases with a similarly increased risk (OR 5.25 (2.75–10.05)). The risk of severe disease associated with the burden of rare *TLR7* variants would likely increase even more if the analyses would have been focused on men, especially those without comorbidities. Moreover, it has been proposed that more common, lower effect size *TLR7* variants could contribute to the male sex bias that is observed in severe COVID-19, because of its innate immune function and X-chromosomal localization [95]. *TLR7* is one of the few genes that escapes X-inactivation, thereby leading to a difference in *TLR7* dosage between men and women [96]. Women exhibit higher basal TLR7 expression levels and more pronounced TLR7-mediated IFN-I responses that aid in viral clearance [96–98]. On the other end of the spectrum, the higher TLR7 dosage in women leads to a predisposition to develop autoimmune disease such as systemic lupus erythematodes (SLE) [99]. Most recently, a *TLR7* de novo missense variant, reported in a female patient with SLE, was shown to cause systemic B-cell-driven autoimmunity through enhanced TLR7 signalling with a break of central B cell tolerance and accumulation of CD11c+ age-associated B cells and germinal centre B cells in mice [100]. Although it is likely that the intrinsic difference in TLR7 dosage between men and women is part of the explanation for the male sex bias in severe COVID-19, the possible additional effect of common genetic variation in *TLR7* should be further investigated.

X-linked TLR7 deficiency has been identified as the first novel immunodeficiency with an isolated, increased susceptibility to severe or critical SARS-CoV-2 infection and has established TLR7 as a critical mediator of IFN-I immunity against SARS-CoV-2. X-linked TLR7 deficiency is expected to account for approximately 1% of cases of severe or critical COVID-19 in men aged under 60 years of age, which is expected to be even higher with stricter screening criteria [91].

### Inborn errors of immunity associated with MIS-C

The prevalence of severe or critical pneumonia in children and young adults is extremely rare [101]. However, in April 2020, a distinct life-threatening complication after SARS-CoV-2 exposure was first described in individuals younger than 21 years and defined as MIS-C [102]. MIS-C typically develops 2–6 weeks after SARS-CoV-2 infection and is characterized by a multiorgan, hyperinflammatory response with fever and elevated inflammatory marker levels [103]. Since MIS-C mostly affects patients that experience only mild or no symptoms during the acute SARS-CoV-2 infection, it is considered to be a postinfectious syndrome that shows similarities with Kawasaki’s disease. The hyperinflammatory immune responses in MIS-C patients have been shown to be distinct from those with acute COVID-19 and Kawasaki’s disease and are associated with pronounced T cell expansion and the production of auto-antibodies [104, 105].

The presence of monogenic IEI predisposing to MIS-C has been investigated in a cohort of 18 children that met the diagnostic criteria for MIS-C. In two boys without previous medical history, rare mutations were identified in the X-linked inhibitor of apoptosis (*XIAP*) and X-linked Cytochrome B-245 Beta Chain (*CYBB*), respectively. The *XIAP* variant was shown to lead to LoF through decreased negative control of the NLRP3 inflammasome, leading to elevated production of pro-inflammatory cytokines in patient CD14^+^ monocytes and PBMCS. Patients with hemizygous LoF mutation in XIAP have been previously shown to be at risk for virally triggered haemophagocytic lymphohistiocytosis (HLH), reminiscent of the hyperinflammation observed in MIS-C [106]. In neutrophils extracted from the patient with *CYBB* mutation, the oxidative burst required for effective phagocytosis and suppression of type I IFN signalling was decreased, thereby providing a mechanism for hyperinflammation [54]. Interestingly, a MIS-C patient was diagnosed with Suppressor Of Cytokine Signaling 1 (SOCS1) haploinsufficiency in a separate study, leading to increased type I and II interferon signalling in unstimulated PBMCs [94]. Transcriptome analysis of unstimulated PBMCS 7 months after recovery demonstrated that the differentially expressed genes of the three patients with a genetic diagnosis were still enriched for inflammatory signalling pathways such as type I IFN signalling compared to other MIS-C patients and patients with mild COVID-19 [54].

Although the pathophysiology of MIS-C is still incompletely understood, these findings in a small group of patients together with the immunological insights suggest that the genetic predisposition to MIS-C is likely to be different from severe COVID-19, impacting the normal control of the immune system.

## Convergence of genetic findings on the interferon signalling pathway

**Fig. 1:**
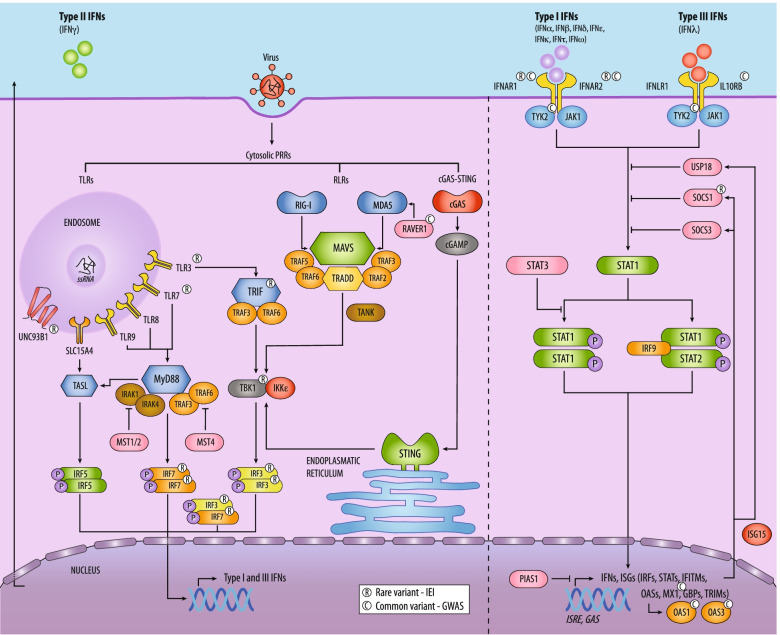
A schematic representation of interferon signalling with display of current genetic findings. The left section of the figure points out the three major cytosolic pattern recognition receptor (PRR) signalling pathways that recognize viruses and culminate in the production of defensive type I and III interferons (IFNs). These routes consist of Toll-like receptor (TLR), RIG-I-like receptor (RLR) and cGAS-STING signalling pathways that utilize distinct adaptor complexes with associating kinases and ubiquitin ligases for their signal transduction. These TASL, MyD88, TRIF and MAVS complexes subsequently lead to the phosphorylation of interferon regulatory factors (IRFs) that initiate transcription of IFNs. Furthermore, the production of the type II IFN interferon gamma (IFNγ) is induced through TLR7-IRF7-dependent signalling. The right section shows autocrine and paracrine signalling of type I and III IFNs through the respective IFNAR1/2 and IFNLR1/IL10RB receptors. This activation leads to the formation of either STAT1 homo- or STAT1/2 heterodimers that recruit IRF9 to induce transcription of IFNs and a plethora of interferon-stimulated genes (ISGs). Several inhibitory proteins are highlighted in pink to illustrate a selection of the negatively regulating feedback loops in this highly regulated pathways. Lastly, symbols above selected proteins indicate whether rare or common variants have been identified in the genes from which these proteins are encoded. *P*, phosphatase; *STAT*, signal transducer and activator of transcription; *IFITM*, interferon-induced transmembrane protein; *OAS*, oligoadenylate synthase; *MX1*, interferon-induced GTP-binding protein; *GBP*, guanylate-binding protein; *TRIM*, tripartite motif protein; *ISRE*, interferon-stimulated response element; *GAS*, gamma-activated sequence

The studies on rare variants in known and novel IEI genes that were discussed in the previous section have highlighted the critical role of IFN-I signalling in the pathogenesis of critical COVID-19. Moreover, several of the GWAS associations implicate common variants that affect genes involved in interferon signalling. The findings from common and rare variant studies that converge on the interferon pathways have been summarized in Fig. 1. This key role for IFN-I signalling in the host defence against SARS-CoV-2 is demonstrated by various other studies [77–79, 107]. The findings from these studies indicate that a delayed induction of the IFN-I response by SARS-CoV-2 rather than its overproduction or complete absence precedes severe COVID-19 [80]. In this interpretation, an early robust IFN response is protective, while a delayed IFN response at a disease stage with high viral loads such as in older hosts fails to limit viral load and leads to inappropriately high circulating interferon (and other inflammatory mediators) concentrations that drive inflammation and collateral organ damage. Evidence for this delayed IFN-I response to SARS-CoV-2 is further corroborated by transcriptomic and protein expression data, which have shown that SARS-CoV-2 induces a low type I (and type III) interferon-stimulated gene (ISG) response, especially when compared with the milder HCoV-229E strain and other respiratory viruses such as influenza [79]. Moreover, SARS-CoV-2 elicits attenuated transcriptional responses of other crucial innate immunity pathways including IL-1 signaling and inflammasome activation [108]. In part, this is due to the broad immune-evasion mechanisms that SARS-CoV-2 employs to antagonize the IFN-I response at the level of virus sensing, IFN signalling and IFN production [80, 109, 110]. Specific examples include impaired recognition of the virus by the cytosolic RIG-I-like receptor (RLR) MDA5, inhibition of STAT1 phosphorylation and blocked translocation of both IRF3 and STAT1 to the nucleus [109, 111]. This interference of SARS-CoV-2 with IFN-I signalling could explain why certain pathways become less redundant or essential, such as the TLR7 pathway.

The relevance of intact early IFN-I signalling is further supported by the discovery of neutralizing auto-antibodies directed against IFN-I in a significant proportion of patients with critical COVID-19 [112]. The prevalence of neutralizing IFN-I auto-antibodies ranged from ~20% in deceased patients, 3–18% in patients with critical COVID-19 pneumonia (in part depending on the concentration of IFN-I used in the neutralization assay) and approximately 7% in patients with severe disease, while auto-antibodies were more uncommon in individuals with asymptomatic or mild infection [113–119]. These auto-antibodies predominantly target the type I IFN subtypes IFNα2 and IFNω but also IFNβ and have been replicated in various other cohorts. Patients with auto-antibodies neutralizing IFN-I had decreased circulating IFNα plasma concentrations [112], impaired expression of IFN-stimulated genes (ISGs) in the nasopharyngeal mucosa and delayed viral clearance [120]. Moreover, anti-IFN-I auto-antibodies were found much more often in men and correlate with infection fatality rates across all ages [121]. These neutralizing IFN-I auto-antibodies therefore constitute both strong determinants and predictors of critical COVID-19 and are considered to be phenocopies of inborn errors of type I IFN immunity. However, it remains unclear whether genetic predisposition underlies the formation of these auto-antibodies and to what extent the auto-antibodies pre-exist or are formed during the SARS-CoV-2 infection as a result of a polyreactive B cell response [115, 122]. Longitudinal measurement suggests that auto-antibodies are formed de novo or can be pre-existing and triggered during SARS-CoV-2 infection [115, 123]. Patients with autoimmune polyendocrine syndrome type 1 (APS-1), who have a defect central T cell tolerance and are consequently prone to develop autoimmune disease due to biallelic germline *AIRE* mutations, have been shown to produce high titres of IFN-I auto-antibodies [124]. One study suggested that APS-I patients with pre-existing auto-antibodies targeting IFN-I were at an increased risk of severe or critical COVID-19, although another study observed only mild symptoms [125]. Furthermore, it has been reported that patients with systemic lupus erythematodes (SLE), Sjögren’s syndrome, RAG1 and RAG2 deficiency and X-linked immunodysregulation polyendocrinopathy enteropathy (IPEX) can generate type I IFN auto-antibodies [126–128]. Further studies are needed to evaluate the risk of pre-existing IFN-I auto-antibodies for the development of severe or critical COVID-19 and the potential role of genetic predisposition.

Since the early host immune response against SARS-CoV-2 is heavily dependent on intact IFN-I signalling, individuals with genetic susceptibility or partial or complete deficiencies in the type I (and possibly the less studied type III) IFN signalling pathways form a group that are at an inherent risk of developing severe or critical disease. This risk may differ based on specifics of the individual variant, including the gene affected, mutation type, genetic ancestry, penetrance and inheritance mode [48]. As an example, a common intronic variant (rs2236757) in *IFNAR2* has a calculated OR of 1.28 to develop critical illness, while the OR is 9 for a rare heterozygous variant in the same gene [32, 48, 51, 82]. We expect that more risk factors at the population level impacting interferon signalling will be identified that could increase susceptibility or confer protection, as well as patients with monogenic IEI in the over 400 other genes related to interferon signalling [129].

## Clinical implications

### Diagnostic sequencing

**Fig. 2:**
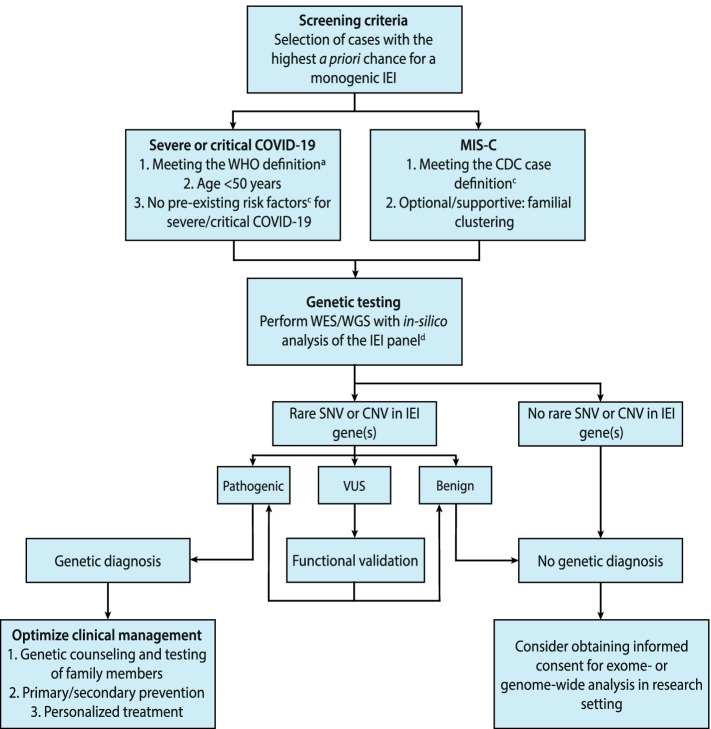
Clinical screening criteria for the implementation of genetic testing to discover rare host genetic factors predisposing to severe/critical COVID-19 or MIS-C. This flowchart proposes genetic screening criteria and a strategy for genetic testing in patients with severe forms of COVID-19 that are suspected of having an underlying IEI. ^a^The diagnostic clinical criteria for severe or critical COVID-19 have been defined according to the WHO definition [133]. ^b^Risk factors that have been associated with severe or critical COVID-19 include chronic comorbidities such as hypertension, diabetes mellitus, obesity (BMI ≥30kg/m^2^), heart failure, chronic lung disease and chronic kidney disease. ^c^The diagnostic clinical criteria for MIS-C have been defined according to the CDC case definition [134]. ^d^The in silico IEI gene panel should contain the genes listed by the most recent update of the International Union for Immunological Societies [67]

Genomic information is increasingly being incorporated in clinical decision-making as next-generation sequencing approaches have improved diagnostic yield, throughput, turnaround time and cost [130–132]. The IEI affecting the TLR3- and TLR7-signalling pathways established so far could already explain up to 5% of critical COVID-19 cases in men under the age of 60 years [51, 120]. When applying stricter screening criteria for age and comorbidities than the inclusion criteria that were used in these previous cohort studies, the prevalence could be higher, such as has been shown for the application of genetic screening for rare *TLR7* variants in a small case series (Table 3) [91]. Since a genetic diagnosis could not only have consequences for the clinical management of the patients but also for affected family members that are still at risk to develop critical disease, we would suggest to screen selected patients suspected of having an underlying IEI. We have created a flowchart that proposes genetic screening criteria and outlines a strategy for genetic testing and follow-up of its results (Fig. 2). This flowchart is intended to create awareness among and offer guidance to the treating physicians of these patients, often internist-infectiologists or intensivists, as well as to other physicians that encounter retrospective cases with a suspect medical history. Moreover, the strategy for genetic testing could help (clinical) geneticists and the treating physicians with the interpretation and validation of the results and possible implications for clinical management.

Diagnostic genetic testing should be considered in cases with the highest probability of having a rare genetic factor that strongly predisposes to severe/critical COVID-19 or MIS-C. This includes relatively young patients <50 years of age who contracted severe or critical COVID-19 as defined according to the WHO definition [133] in the absence of pre-existing risk factors, and all patients with MIS-C that meet the CDC case definition criteria [134]. In the selected patients, a targeted in silico IEI gene panel analysis could be performed after WES or WGS to identify rare, non-synonymous single-nucleotide variants (SNVs) or copy number variants (CNVs). The IEI panel should include the latest IEI gene list of the International Union of Immunological societies that includes the genes described above and summarized in Table 3 and can be easily updated when novel genes are discovered, with limited risk of incidental findings [67]. Rare variants that are identified in IEI genes should be classified according to the ACMG criteria [135]. Variants of unknown significance (VUS) should prompt functional validation experiments to support pathogenicity. Heterozygous variants in recessive IEI genes should not be disregarded, as relatively mild functional consequences at the protein level that would normally not lead to disease could still be relevant in the interaction with specific pathogens. This difference in redundancy is for example implied by the heterozygous variants found in recessive IFN-I genes in patients with critical COVID-19 [51], but also those found in recessive HLH-genes that were enriched among patients with severe manifestations of COVID-19 [136]. Most recently, it was demonstrated that haploinsufficiency of OTULIN due to heterozygous variants was associated with an increased risk of life-threatening necrosis upon staphylococcal infection, while complete OTULIN deficiency causes an auto-inflammatory syndrome [137]. Similarly, hypomorphic variants that only partially reduce protein function in dominant IEI genes could still exert a significant effect.

If no rare variants of interest are found in IEI genes, it should be contemplated to obtain informed consent for an exome- or genome-wide analysis after counseling by a clinical geneticist to explore novel genes associated with severe/critical COVID-19 or MIS-C. Although many of the genetic variants identified so far converge on genes involved in interferon signalling, it would be of interest to investigate other pathways, for example those involved in virus entry, intrinsic immunity by the pulmonary epithelial cells and control of the immune response. The importance of the pulmonary epithelial cells in the host defence against SARS-CoV-2 is underlined by common variants implicating genes involved in their function that were enriched in severe COVID-19, which have been discussed earlier. Moreover, genetic variants that increase IL-6 signalling could be of interest. Elevated IL-6 serum concentrations are a hallmark of severe disease and although circulating plasma IL-6 levels are lower compared to other causes of ARDS, treatment with the anti-IL-6 drug tocilizumab has proven effective [138–140]. Also, genetic variants that impact the normal control of the immune response that could lead to immune dysregulation or hyperinflammation might increase the risk for severe manifestations of COVID-19, including MIS-C.

Another clinical application that could leverage the generated genomic data is the development of a polygenic risk score (PRS). The PRS is emerging as an increasingly reliable risk predictor in some diseases such as breast cancer [141]. In COVID-19, a PRS could estimate the individual risk for disease susceptibility or severity based on common and rare variants that are found to be enriched in GWAS studies. Although some studies have already attempted to model the PRS for COVID-19, more research is required to build a PRS model that is robust and can be applied to patients with different genetic and medical backgrounds [41, 50, 142, 143].

### Clinical management of inborn errors of type I IFN signalling

When a genetic diagnosis is established, this could have consequences for clinical management. Some diagnostic centres have implemented rapid exome sequencing (rWES) in clinical care, which may provide a diagnosis within one week [8, 132]. The original identification of X-linked TLR7 deficiency by rapid clinical WES demonstrates that this technique could be implemented in a clinical setting as a diagnostic tool [8]. In a minority COVID-19 cases, this could enable a quick diagnosis that might alter therapeutic management in later stages of the disease. However, in most diagnostic centres, this will not be a feasible approach. The strongest benefit of a diagnosis will therefore reside in the opportunity to take preventative measures, including strict adherence to the current protective measures that are advised by the local government, appropriate vaccination strategy, a direct line of communication to the hospital and pre-emptive hospitalization for clinical observation with early initiation of treatment. A genetic diagnosis could also identify pre-symptomatic family members that have a deficiency of IFN-I signalling. Although vaccination has significantly reduced the risk of developing critical COVID-19, novel SARS-CoV-2 variants could emerge that (partly) escape the immunological memory and would be especially dangerous to patients with IEI of IFN-I immunity. The identification of male hemizygous *TLR7* mutation carriers is particularly relevant given the high clinical penetrance for severe COVID-19, as only few asymptomatic carriers have been reported so far [53, 91]. Genetic counselling and testing of the family members at risk would enable an option for primary prevention and personalized treatment in case of SARS-CoV-2 infection.

In the situation that carriers are infected, they should be hospitalized at an early stage of infection. The same approach could be taken in patients that are re-infected with SARS-CoV-2, although these infections are expected to be limited in severity as patients should develop a normal humoral immune response. Type I interferon treatment might have a rationale in patients with partial or complete IFN-I deficiencies, as it could replenish the shortage of IFN-I and thereby limit viral replication and secondary inflammation. However, there is currently no convincing support for the administration of exogenous IFN-I in COVID-19, with some clinical trials showing a beneficial effect [144–148] while other, larger trials did not [149, 150]. These studies differ in patient population, route of administration, outcomes and initiation of treatment after disease onset, although substantial evidence suggests that early timing of treatment is key [80, 151]. Despite this ongoing debate, patients with inborn errors of IFN-I production are expected to respond better to treatment with type I interferon than the average COVID-19 patient with a presumed sufficient IFN-I production capacity. Illustratively, in two patients with IFN-I deficiency (autosomal dominant TLR3 and IRF3 deficiency) who developed severe COVID-19, it was shown that treatment with a single dose of recombinant IFNα2a could resolve symptoms within 48 h [152]. More studies are however required to investigate the place of recombinant IFN-I therapy for IEI patients. Since the prevalence of IEI of IFN-I signalling are estimated to affect up to 5% of patients with critical COVID-19, it would be informative to test patients enrolled in such studies for genetic (or immunological) IFN-I deficiency to investigate the therapeutic benefit of IFN-I therapy in this specific patient group.

Since patients with X-linked TLR7 deficiency and possibly also patients with other IFN-I deficiencies have a defective type II interferon production in response to SARS-CoV-2, recombinant interferon gamma (IFNγ) could be an alternative treatment option [153]. Some experience with this drug has been gained in patients with chronic granulomatous disease (CGD), an immunodeficiency in which treatment or prophylaxis with IFNγ is used to treat or prevent infections, respectively [154]. Moreover, treatment with recombinant IFNγ was shown to be beneficial for SARS-CoV-2 clearance in patients with prolonged detectable virus [153].

In summary, diagnostic screening for rare variants should be considered in selected patients with severe manifestations of COVID-19. A genetic diagnosis could enable more personalized management of the patient as well as genetic counseling of family members that are at risk. Recognition of patients with predisposing rare variants or IEI remains important even though most individuals are protected by vaccination or prior exposure, since it is uncertain whether novel SARS-CoV-2 escape variants might arise with high pathogenicity. Lastly, targeted treatment with recombinant IFN-I might be a feasible option that should be investigated in future studies.

## Conclusions

The discovery of known or novel monogenic IEIs that confer predisposition to severe COVID-19 can give important insights into the immunopathogenesis of SARS-CoV-2 infection. GWAS studies have generated important associations between genetic loci and the pathogenesis of SARS-CoV-2 infection at the population level. Although these associations may provide polygenic risk scores or ultimately inform development of future therapeutic approaches, they have not yet provided actionable information for the individual patient. It might be of interest to study the interplay of rare and common genetic variation for both the susceptibility to and protection against SARS-CoV-2 infection. Although monogenic IEI are individually rare, collectively, they could account for a significant percentage of severe COVID-19 cases as illustrated by the identification of inborn errors of type I interferon signalling. Of these, X-linked TLR7 deficiency is the most robustly replicated and could account for at least 1% of critical cases in men. The knowledge gained from the study of monogenic IEI could directly benefit predominantly young, previously healthy patients with severe COVID-19 and possibly their relatives, by providing a genetic diagnosis through the implementation of WES or WGS in clinical care. Such a diagnosis could create a rational basis for clinical counselling, preventative measures and possibly therapeutic interventions.

## Data Availability

Data sharing is not applicable to this article as no datasets were generated or analysed during the current study.
